# Early and Late Direct Costs in a Southern African Antiretroviral Treatment Programme: A Retrospective Cohort Analysis

**DOI:** 10.1371/journal.pmed.1000189

**Published:** 2009-12-01

**Authors:** Rory Leisegang, Susan Cleary, Michael Hislop, Alistair Davidse, Leon Regensberg, Francesca Little, Gary Maartens

**Affiliations:** 1 Division of Clinical Pharmacology, Department of Medicine, University of Cape Town, Cape Town, South Africa; 2 Health Economics Unit, School of Public Health and Family Medicine, University of Cape Town, Cape Town, South Africa; 3 Aids for AIDS, Medscheme, Cape Town, South Africa; 4 Department of Statistical Sciences, University of Cape Town, South Africa; Boston University, United States of America

## Abstract

**Background:**

There is a paucity of data on the health care costs of antiretroviral therapy (ART) programmes in Africa. Our objectives were to describe the direct heath care costs and establish the cost drivers over time in an HIV managed care programme in Southern Africa.

**Methods/Findings:**

We analysed the direct costs of treating HIV-infected adults enrolled in the managed care programme from 3 years before starting non-nucleoside reverse transcriptase inhibitor-based ART up to 5 years afterwards. The CD4 cell count criterion for starting ART was <350 cells/µl. We explored associations between variables and mean total costs over time using a generalised linear model with a log-link function and a gamma distribution. Our cohort consisted of 10,735 patients (59.4% women) with 594,497 mo of follow up data (50.9% of months on ART). Median baseline CD4+ cell count and viral load were 125 cells/µl and 5.16 log_10_ copies/ml respectively. There was a peak in costs in the period around ART initiation (from 4 mo before until 4 mo after starting ART) driven largely by hospitalisation, following which costs plateaued for 5 years. The variables associated with changes in mean total costs varied with time. Key early associations with higher costs were low baseline CD4+ cell count, high baseline HIV viral load, and shorter duration in HIV care prior to starting ART; whilst later associations with higher costs were lower ART adherence, switching to protease inhibitor-based ART, and starting ART at an older age.

**Conclusions:**

Drivers of mean total costs changed considerably over time. Starting ART at higher CD4 counts or longer pre-ART care should reduce early costs. Monitoring ART adherence and interventions to improve it should reduce later costs. Cost models of ART should take into account these time-dependent cost drivers, and include costs before starting ART.

*Please see later in the article for the Editors' Summary*

## Abstract 2

Gary Maartens and colleagues describe the direct heath care costs and identify the drivers of cost over time in an HIV managed care program in Southern Africa.

## Editors' Summary

**Background:**

About 30 million people (22 million people in sub-Saharan Africa alone) are infected with the human immunodeficiency virus (HIV), the cause of acquired immunodeficiency syndrome (AIDS). HIV destroys immune system cells (including CD4 cells, a type of lymphocyte), leaving infected individuals susceptible to other infections. Early in the AIDS epidemic, on average HIV-positive people died within 10 years of infection. Then, in 1996, highly active antiretroviral therapy (ART; combinations of powerful antiretroviral drugs) was developed. For people living in affluent, developed countries HIV/AIDS became a chronic, treatable condition, but for the millions of HIV-infected people living in low- and middle-income countries, effective treatment was unavailable and HIV/AIDS remained a fatal illness. In 2003, this situation was declared a global health emergency and governments, international agencies, and funding bodies began to implement plans to increase ART coverage in developing countries. By the end of 2008, of the 9.5 million people in need of ART in low- and middle-income countries, more than 4 million people were receiving treatment.

**Why Was This Study Done?:**

Good progress is being made towards achieving universal access to ART, partly because the cost of antiretroviral drugs has plummeted in developing countries. But the provision of antiretroviral drugs is not the only direct cost associated with ART. General practitioner, specialist, and maternity-related care for patients receiving ART, hospital accommodation when necessary, and the investigations that are needed to monitor the progress of HIV infection such as CD4 cell counts and viral load measurements all incur considerable costs. To use their limited resources effectively, public-health officials in developing countries need to know as much as possible about the direct costs of HIV health care but few studies have investigated these costs, particularly those incurred before an individual starts taking ART. In this study, the researchers explore health care costs in a South African private-sector HIV/AIDS program and examine the variables that drive the costs of HIV health care around the time of ART initiation and during later phases of ART.

**What Did the Researchers Do and Find?:**

The researchers analyzed the direct costs of treating more than 100,000 HIV-infected adults enrolled in a private HIV care program in South Africa from 3 years before they started ART until up to 5 years after ART initiation; within this program, individuals began to receive ART when their CD4 cell count fell below 350 cells/µl of blood. The researchers found a peak in direct health costs from 4 months before to 4 months after starting ART (the “peri-ART” period), which was driven mainly by hospital costs. After the peri-ART period, costs dropped (although not to the levels seen before this period) and stabilized at an intermediate level for the next 5 years. Detailed statistical analyses suggest that the key variables associated with higher costs in the peri-ART period were a low baseline CD4 cell count, a high baseline HIV viral load, and a shorter time in HIV care before ART initiation. The key variable associated with higher costs later in ART was lower adherence to the drug therapy. That is, costs were higher among patients who did not take their antiretroviral drugs regularly.

**What Do These Findings Mean?:**

This study involved patients enrolled in a private health care program in which the criteria for initiating ART differed somewhat from those recommended by the World Health Organization for ART initiation in resource-limited settings. Thus, the absolute mean total costs calculated by the researchers are unlikely to be generalizable to public HIV care systems in South Africa and in other resource-poor settings. However, the finding that the drivers of mean total costs change considerably over time may be generalizable and provides some useful information for public-health planners that can now be tested in other, more resource-limited patient populations. In particular, the findings of this study suggest that the high early costs of ART programs could be reduced by starting ART at higher CD4 cell counts or by providing longer pre-ART care. In addition, the findings suggest that monitoring ART adherence and introducing interventions to improve ART adherence could reduce the later direct costs of ART programs.

**Additional Information:**

Please access these Web sites via the online version of this summary at http://dx.doi.org/10.1371/journal.pmed.1000189.

- Information is available from the US National Institute of Allergy and infectious diseases on HIV infection and AIDS
- HIV InSite has comprehensive information on all aspects of HIV/AIDS
- Information is available from Avert, an international AIDS charity on many aspects of HIV/AIDS, including information on the HIV and AIDS in Africa, and on universal access to AIDS treatment (in English and Spanish)
- The World Health Organization provides information about universal access to AIDS treatment, including the September 2009 progress report (in English and French)
- The US Centers for Disease Control and Prevention also provides information on global efforts to deal with the HIV/AIDS epidemic

## Introduction

Access to combination antiretroviral therapy (ART) is rapidly expanding in resource-limited settings. Data on the costs of providing HIV health care and how these change over time are important for guiding resource allocation. However, there are few good quality studies of the direct health care costs of HIV infection, as illustrated by a recent systematic review that found only nine studies from the ART era that fulfilled inclusion criteria [1]. Data on costs prior to starting ART are limited as most cost studies only report costs once ART has been commenced. A recent South African study reported that health care costs were almost twice as high in the first year on ART in comparison with the second year [2]. However, the sample size was small, patients had advanced disease, follow up was relatively short, and the period of higher costs in the first year on ART was not defined.

Delays in establishing public ART programmes in South Africa until 2003 [3], together with studies highlighting the detrimental effects of HIV in the workplace [4], resulted in the scaling up of access to ART through private medical insurance funds from as early as 1998 [5]. Given the level of need for improved access to ART in South Africa, which has the world's largest number of HIV-infected people [6], the government has identified partnerships with the private sector as a key mechanism for enhancing access [7]. In the private sector, starting ART is encouraged earlier than current WHO guidelines for resource-limited settings [8], thus enabling exploration of the cost implications of starting ART earlier.

The objective of this study was to explore health care costs in a South African private sector HIV/AIDS programme, with a special focus on the determinants of costs around the period of ART initiation, as well as the determinants of costs during the later phases of ART.

## Methods

### Ethics Statement

The study was approved by the Research Ethics Committee, University of Cape Town and by the Board of Directors of Aid for AIDS (AfA). Informed consent was not required as the data were analyzed anonymously, but all patients signed consent for their information to be entered into the AfA database.

### Data Source

Data for this study were extracted from a database of patients enrolled with AfA, a group that manages HIV-related care for a number of medical insurance funds and companies in the private sector in Southern Africa [9]. Registration of eligible patients with AfA is done by the private doctor looking after the individual patient (i.e., there are no clinics, but some private doctors run exclusive HIV practices). Demographic data, CD4+ cell count, viral load, and previous ART history is captured centrally. Patients are managed according to a clinical guideline and any decision to start ART, change ART regimen, and treat certain opportunistic infections is subject to review and approval by AfA clinical staff. The antiretroviral guidelines are similar in many respects to WHO guidelines for resource-poor settings [8] and the South African public sector programmes, but ART is initiated earlier and there is room for choice of individual antiretroviral drugs. For example several ritonavir-boosted protease inhibitors (PIs) are available rather than the single one available in the South African public sector. ART can be initiated at CD4+ cell counts <350 cells/µl rather than <200 cells/µl in the South African public sector, but similar to WHO guidelines that recommend initiation with CD4+ cell counts <350 cells/µl with symptomatic disease. The recommended initial regimen is a combination of two nucleoside reverse transcriptase inhibitors (NRTIs) and a non-nucleoside reverse transcriptase inhibitor (NNRTI). Second line therapy consists of a boosted PI with two NRTIs. CD4+ cell counts and viral loads are monitored 6 monthly. There is a telephonic counselling service provided by AfA, although counselling is not routine but done on demand. Drugs are collected monthly from private pharmacies.

### Inclusion Criteria

Two of the medical insurance funds contracted to AfA were selected on the grounds that they had large numbers of patients, similar treatment benefits, and required no co-payment for ART. This selection allowed us to describe costs and drivers of costs without relating to the patient's ability to pay, which has been reported to influence outcomes on ART [10]–[12]. Patients were included in the study if they were ART naïve at entry (women who had received prophylaxis for prevention of mother-to-child transmission were not excluded); adult (19 y or older at the time of approval for ART); and if ART was started between November 1998 and November 2007. Patients were excluded if they had missing cost data over the entire period. To make our findings more generalisable, we only included patients starting ART with an NNRTI plus two NRTIs, as recommended by the WHO for resource-limited settings [8].

### Cost Data

Direct health care costs were analysed from the provider's perspective [13], and indirect costs were not assessed. Submitted health care claims were captured into a central database. The tariff amount (which is the amount agreed to annually following negotiations between private healthcare providers and funders) was used as a proxy for direct health care costs, as opposed to the amount charged by the provider or the amount reimbursed to the patient. Of the 49,517 unique claim categories in the AfA database, 4,000 accounted for over 95% of total costs. These 4,000 claim categories were grouped into the following categories: ART, other medication, maternity-related care (antenatal services, delivery, caesarean section, and postdelivery paediatric care), general practitioner care, specialist care, hospital accommodation and procedures, CD4+ cell count and viral load monitoring, other investigations (e.g., laboratory tests and radiology).

The prices of antiretroviral drugs have fallen dramatically over the period of our study. To account for this decrease we deflated ART prices to the April 2007 level. All other health care costs have increased; these were inflated to the April 2007 level using the consumer price index net of mortgage payments (CPIX) [14]. The average South African rand to US$ exchange rate in April 2007 (R7.14 to US$1) was used to convert costs to US$ equivalents [15].

### Exploratory Cost Analysis

The mean total cost and its components were explored from 36 mo before starting ART to 60 mo on ART. Costs were broken down into the following components: ART, other medication, hospitalisation, investigations, CD4+ cell count and HIV viral load monitoring, general practitioner consultations, specialist consultations, maternity, and auxiliary care. This exploratory analysis revealed a marked peak in cost from 4 mo before starting ART until 4 mo on ART. This 8-mo interval is denoted the “peri-ART” period in this study.

### Statistical Methods

Even though health care costs are often right-skewed, with a minority of patients incurring very high costs, the health economics literature argues that health care policy decisions are best guided by analyses of arithmetic mean costs, as the mean provides information on the costs of treating the entire population [16]. Thus ordinary least squares (OLS) regression models and generalised linear models (GLM) were considered [16],[17]. The month in which patients started ART was set as the zero month for all patients, which provided a common reference point for all the patients in our analysis. We divided the period from 4 mo before to 60 mo after starting ART into 4-mo intervals and determined the mean total cost in each interval. As many months had zero costs (>10%), using the mean cost over 4 mo intervals resulted in few zero values in the outcome variable, thus avoiding the need for zero-inflated models. A GLM with a gamma distribution and a log-link function was selected on grounds that it could describe the distribution of the data. An OLS model was abandoned because it was unable to adequately account for the patients with very high costs, which was a significant proportion of total costs. With a log-link function, the variables are associated with a proportional change in total mean costs. Improved residual diagnostics, lower Akaike Information Criteria (AIC), and improved trend prediction were used in the model development and refinement.

The time-varying associations between mean total cost and the variables was modelled using three methods: a separate model for each 4-mo time interval using categorical variables, and two models with categorical or continuous variables over the entire interval with time included as a variable, which also interacted with the other variables. Effect estimates and their significance at the 95% level were assessed using robust standard errors with clustering at an individual level. Data storage, basic calculations, and data extraction was handled in Microsoft Access 2003 [18] and statistical analysis was performed in Stata 10 [19].

The following variables were considered in our analysis: baseline CD4+ cell count and HIV viral load (baseline was defined as the most recent result within 6 mo before starting ART), ART adherence assessed by monthly pharmacy claims, age, sex, the NNRTI and the NRTI combination used in patients on first line therapy, whether the patient switched to PI-based second line ART, and the duration of CD4+ cell count monitoring (as a proxy for being in HIV care) prior to starting ART. Patients with less than 4 mo of claims data after starting ART were excluded on the grounds that we were unable to assess their ART adherence over shorter time intervals. We split the continuous variables into the following categories: (1) Baseline CD4+ cell count was divided into four groups: 0–49, 50–199, 200–349, and ≥350 cells/µl. (2) HIV viral load was categorised as ≥100,000 copies/ml or <100,000 copies/ml. (3) The mean ART adherence was determined using pharmacy refill data and divided into quartiles. (4) The NNRTI was included as a binary variable (either efavirenz or nevirapine); whereas (5) the NRTI combination in first line was divided into three groups: zidovudine and lamivudine, stavudine and lamivudine, or any other combination. (6) A binary variable was used to reflect whether or not the patient was on second line ART. (7) Age was divided into quartiles. (8) Sex was included as a binary variable. (9) Patient follow-up for HIV prior to starting ART was measured by the length of time between the first CD4+ cell count and the date of starting ART, and was categorised as less than 6 mo and more than 6 mo.

## Results

10,735 patients met our eligibility criteria. The characteristics of the cohort are described in Table 1. There were almost 600,000 patient months of observation, about half of which were on ART. Median follow-up on ART was 26 mo. Baseline body mass index (BMI) was only available for 4,416 of the patients: 13% were <18.5 kg/m^2^, 52% were ≥18.5 kg/m^2^ and <25 kg/m^2^, and 35% were ≥25 kg/m^2^. The most common first line and second line antiretroviral regimens were zidovudine, lamivudine, and efavirenz and lopinavir/ritonavir, zidovudine, and didanosine, respectively. CD4 and viral load monitoring were done 1.5 times per annum on average. Hospitalisation rates were 441 d per 100 patient years of observation (PYO) in the first 6 mo of ART and 179 d per 100 PYO subsequently. Hospitalisation incidence was highest in patients in the lowest CD4 count stratum.

**Table 1:** The characteristics of the cohort.

Characteristics		Overall *n* = 10,735	Regression Subset *n* = 7,427
**Patient months included in analysis**	**Overall**	594,497	282,141
	**On ART**	302,579 (50.9%)	252,433
**Duration on ART (mo)**	**Median**	26	33
	**IQR**	(9–44)	(16–50)
**Age at starting ART (y)**	**Median**	37	37
	**IQR**	(32–43)	(32–43)
**Sex**	**Female**	6,379 (59.%)	4,557 (61%)
	**Male**	4,356 (41%)	2,897 (39%)
**Patient status at end of study period**	**Active**	6,339 (59%)	4,217 (56%)
	**Left scheme**	3,329 (31%)	2,669 (36%)
	**Dead**	1,067 (10%)	1,067 (8%)
**Baseline CD4+ cell count**	**Median**	125 cells/µl	125 cells/µl
	**IQR**	(49–203)	(55–204)
	**Missing**	1,726	N/A
**Baseline viral load (log10)**	**Median**	5.20	5.16
	**IQR**	(4.70–5.60)	(4.66–5.59)
	**Missing**	2,031	N/A
**NNRTI used in first line**	**Nevirapine**	2,655 (28%)	2,432 (28%)
	**Efavirenz**	6,711 (72%)	6,127 (72%)
**NRTI combination in first line**	**AZT+3TC**	6,950 (65%)	4,945 (67%)
	**D4T+3TC**	1,564 (15%)	1,684 (23%)
	**Other**	2,221 (20%)	798 (10%)
**Duration of CD4+ cell count monitoring before starting ART (mo)**	**Median**	1.5	1.5
	**IQR**	(0.7–4.2)	(0.7–4.2)

3TC, lamivudine; AZT, zidovudine; D4T, stavudine; IQR, interquartile range; N/A, not applicable.

The proportion of patients who left the scheme was 31% overall and 24% at 2 y. Patients who left the scheme either changed their employment, switched to a different medical insurance scheme, or voluntarily stopped their contributions to the insurance scheme. Patients who left the scheme differed from those who did not leave in the following baseline characteristics (established using the Wilcoxon rank sum test for continuous variables and Chi-squared test for categorical variables): viral load (median of 5.2 versus 5.1 log_10_, *p = *0.0016), proportion female sex (57% versus 60%, *p = *0.0001), and age (37.4 y versus 37.0 y, *p = *0.0006). Importantly, these baseline differences are not clinically significant and the CD4+ cell count did not differ significantly (median of 127 versus 123 cells/µl, *p = *0.137).

### Exploratory Cost Analysis

**Figure 1:**
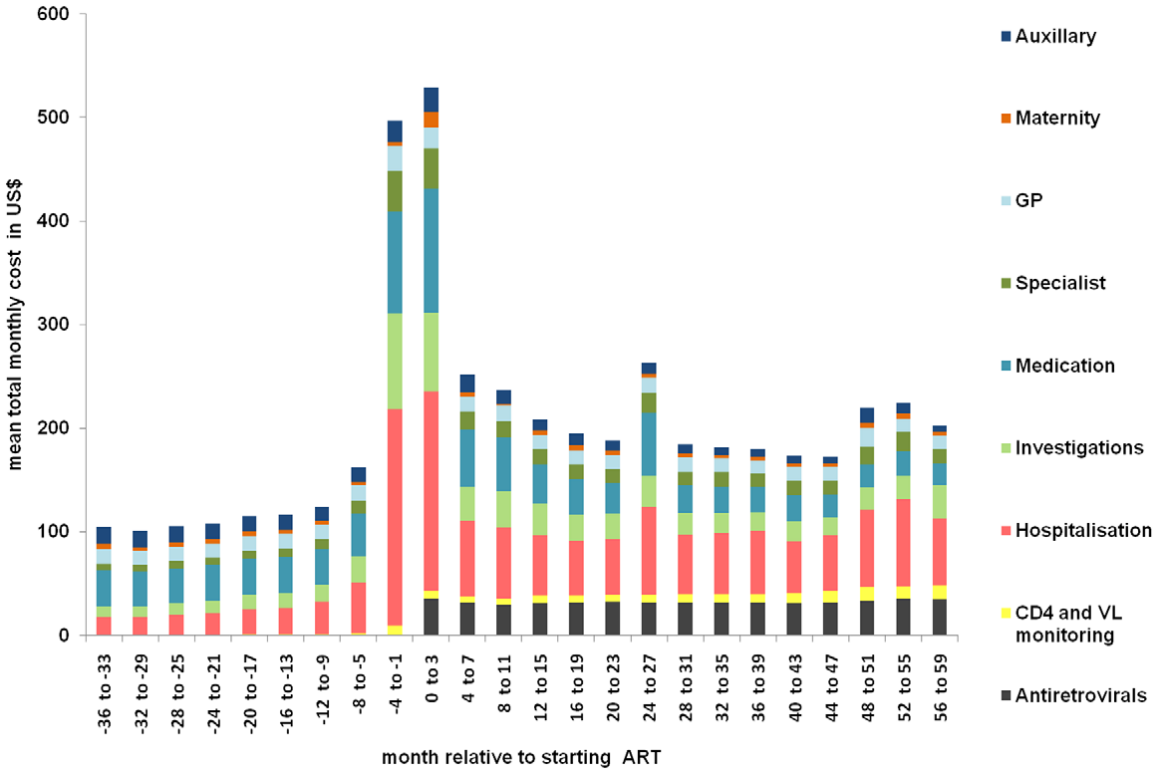
Mean categorised total monthly costs from 36 mo before starting to 60 mo on ART.

The cost data were highly skewed with 10% of the population accounting for 90% of the costs. Figure 1 shows the mean monthly cost and its components. The mean monthly cost rose from a plateau of around US$100 per month before ART, to a peak of around US$500 in the peri-ART period, before dropping down to a new higher plateau of around US$200 on ART. Median (IQR) monthly costs are shown in Figure S1.

### Multiple Regression Analysis

After excluding patients with missing demographic and baseline viral load and CD4+ cell count data, 7,427 patients were included in this analysis, and their characteristics are shown in Table 1. The summary statistics for this subset were comparable with the full dataset.

**Figure 2:**
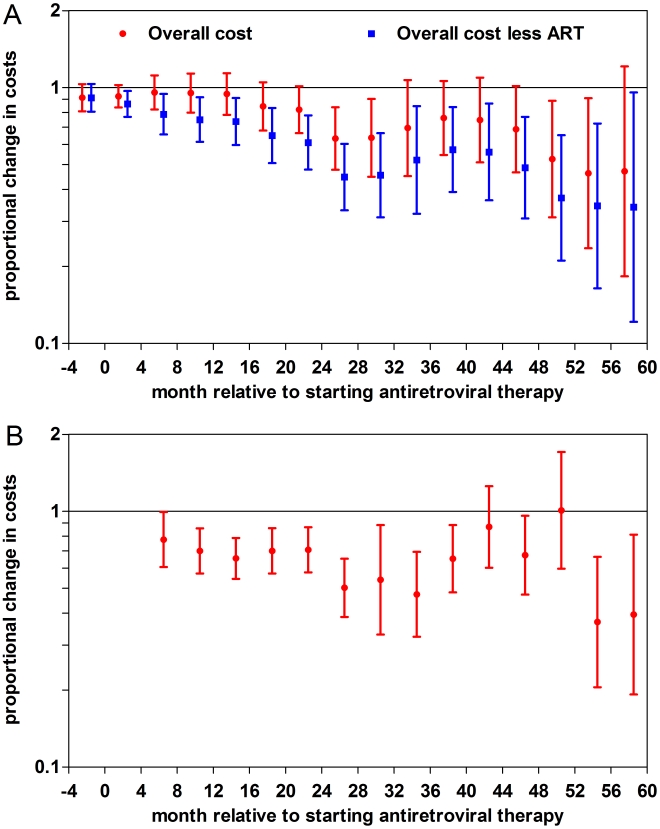
The proportional change in mean total costs associated with ART adherence with 95% confidence intervals. (A) The highest overall ART adherence quartile was compared with the lowest adherence quartile within each time interval (ART costs included and excluded) from 4 mo before starting ART to 60 mo on ART. (B) The highest lagged ART adherence group was compared with the lowest group (≥3 monthly versus ≤1 monthly refills in the previous 4-mo period) within each time interval from 4 mo before starting ART to 60 mo on ART.

In our first analysis, we modelled each 4-mo time interval separately. We found that lower baseline CD4+ cell counts and high HIV viral loads were associated with increased mean total cost predominantly from 4 mo before to 8 mo after starting ART. In contrast, the highest ART adherence quartile was increasingly associated with lower mean total cost over time when compared with the lowest quartile (Figure 2A). When ART-related costs were excluded (on the grounds that high adherence would result in more ART-related costs), the association was more marked. In a subanalysis, the effect of lagged ART adherence (adherence in the prior 4 mo) on costs per 4-mo period was assessed. Again higher adherence in the prior 4 mo was associated with lower costs (Figure 2B). Being on second line (PI-based) ART was associated with higher costs throughout the time period. The other variables were associated with small effects (<10%), which were largely not significant. In a subanalysis, we excluded maternity (which is associated with high costs as nearly all women deliver by caesarean section) and ART costs (a lower proportion of women started efavirenz, which is teratogenic and more expensive than nevirapine) and found the association between sex and mean total cost was not consistent and marginal (<10%).

Mean total costs fell over the first 24 mo on ART and thereafter cost remained constant. Similarly, the associations between many variables and mean total costs changed over the first 24 mo and thereafter remained constant. We found that splitting time into four periods (−4 to 4 mo, 5–12 mo, 13–24 mo, and >24 mo) described time-dependent association between time and total mean cost and its interaction with the other variables. The results from this multiple regression analysis are found in Table 2.

**Table 2:** The proportional change in mean total cost modelled using a multiple generalised linear model regression.

Variable		Time Intervals (mo)					
		−4 to 4	5–12		13–24		>24
**Mean monthly total cost (US>$)**	**—**	377 (337–418)	183 (160–206)		161 (138–183)		115 (98–131)
**Baseline CD4 count (cells/µl)**	**<50**	1.98 (1.74–2.22)	1.35 (1.07–1.63)		1.28 (1.05–1.51)		1.23 (0.97–1.48)
	**50–199**	1.34 (1.20–1.48)	1.08 (0.94–1.21)		1.02 (0.87–1.17)		1.31 (1.11–1.51)
	**200–349**	1 (referent)	—		—		—
	**≥350**	1.57 (1.21–1.92)	1.39 (0.97–1.8)		1.43 (0.96–1.89)		1.12 (0.78–1.45)
**Baseline viral load (copies/ml)**	**≥100,000**	1.24 (1.10–1.37)	1.08 (0.93–1.23)		1.08 (0.94–1.23)		1.09 (0.95–1.23)
	**<100,000**	1 (referent)	—		—		—
**Age at starting ART (y)**	**<25**	1.03 (0.81–1.26)	0.82 (0.64–0.99)		0.83 (0.60–1.06)		0.85 (0.59–1.12)
	**25–49**	1 (referent)	—		—		—
	**≥50**	1.20 (0.97–1.43)	1.14 (0.87–1.42)		1.01 (0.79–1.23)		1.52 (0.72–2.32)
**Sex**	**Male**	0.98 (0.87–1.10)	1.00 (0.83–1.17)		0.91 (0.77–1.06)		0.91 (0.75–1.06)
	**Female**	1 (referent)	—		—		—
**NNRTI**	**Nevirapine**	0.87 (0.77–0.96)	0.89 (0.78–1.00)		1.11 (0.96–1.27)		1.02 (0.86–1.18)
	**Efavirenz**	1 (referent)	—		—		—
**NRTI combination**	**D4T/3TC**	1.05 (0.88–1.22)	1.01 (0.80–1.22)		0.95 (0.74–1.16)		0.96 (0.61–1.32)
	**Other**	0.91 (0.81–1.02)	0.98 (0.82–1.14)		1.05 (0.88–1.22)		1.06 (0.84–1.27)
	**AZT/3TC**	1 (referent)	—	—			—
**Duration CD4+ cell count monitoring**	**≥6 mo**	0.76 (0.67–0.85)	0.98 (0.84–1.12)	1.01 (0.87–1.16)			1.30 (1.06–1.54)
	**<6 mo**	1 (referent)	—		—		—
**Therapy**	**Second line**	1.65 (1.09–2.2)	3.10 (0.43–5.76)	1.94 (1.45–2.44)			2.06 (1.53–2.58)
	**First line**	1 (referent)	—	—		—	
**Mean overall ART adherence**	**<38%**	0.84 (0.75–0.94)	1.00 (0.86–1.14)	1.17 (0.98–1.35)		1.54 (1.21–1.86)	
	**38%–73%**	1.08 (0.97–1.20)	1.25 (1.01–1.49)	1.12 (0.97–1.27)		1.28 (1.07–1.50)	
	**74%–92%**	0.85 (0.77–0.94)	1.25 (1.06–1.44)	1.06 (0.92–1.21)		1.09 (0.92–1.26)	
	**>92%**	1 (referent)	—	—		—	

A log-link function with a gamma distribution was used in the model. Numbers in parentheses are the 95% confidence intervals. 3TC, lamivudine; AZT, zidovudine; D4T, stavudine.

We found that costs were very high in the peri-ART period. Mean monthly costs were more than 3 times higher in this period and the association between costs and baseline CD4 count and baseline viral load were more marked in the peri-ART than in later time periods. In the above analysis, we excluded patients who died within the first 4 mo on ART because we could only estimate ART adherence over a period of 4 mo or longer. However given that patients who died might incur significant costs, we performed an additional subanalysis including these early deaths. This subset included 8,559 patients. The findings were similar but the associations between variables and total mean costs diminished marginally when we repeated the multiple regression analysis with the ART adherence variable excluded (unpublished data).

**Figure 3:**
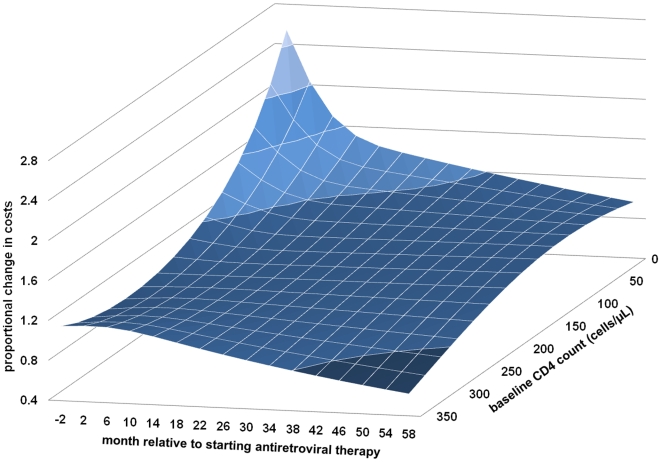
The proportional change in mean total monthly costs over time associated with baseline CD4 cell count. Baseline CD4 count was compared with the referent group (200 cells/µl) within each time interval from 4 mo before starting ART to 60 mo on ART with lighter blue indicating higher relative costs.

**Figure 4:**
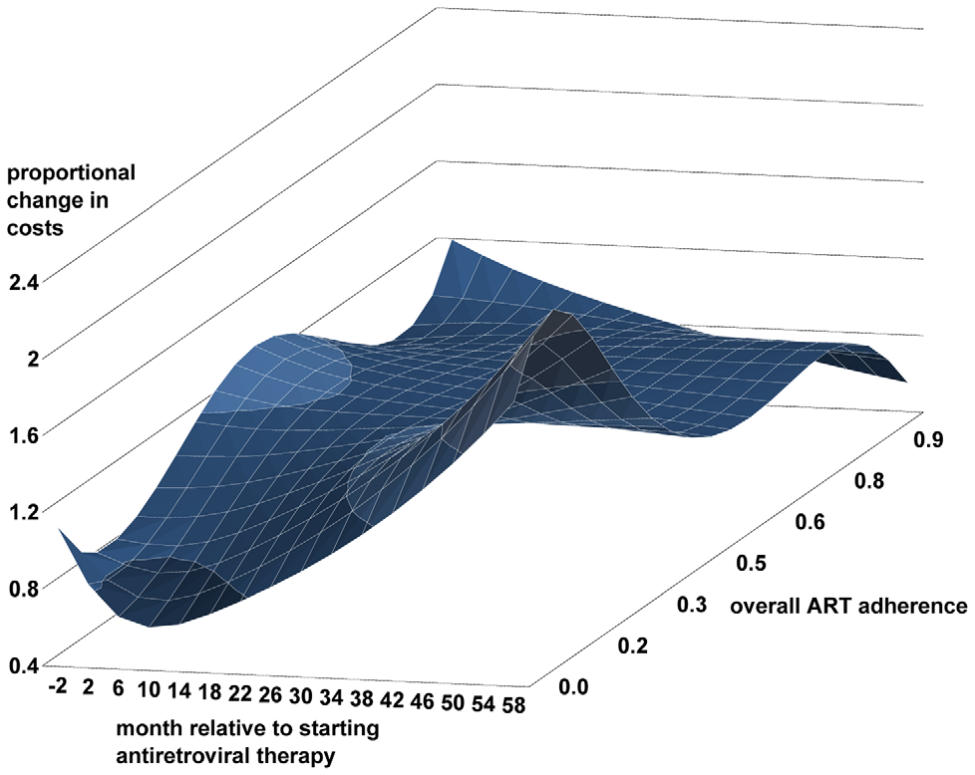
The proportional change in mean total monthly costs over time associated with overall ART adherence. Overall ART adherence was compared with the referent group (75%) within each time interval from 4 mo before starting ART to 60 mo on ART with lighter blue indicating higher relative costs.

Finally, we explored continuous models for ART adherence, age at starting ART, baseline viral load, and baseline CD4+ cell count (only counts <350 cells/µl were analysed as patients with higher counts were started on ART for serious HIV-related morbidity): polynomial functions of the 2nd degree were used for all the variables except ART adherence (4th degree polynomial) and baseline HIV viral load (3rd degree polynomial) were used. We felt that the duration of CD4+ cell count monitoring before staring ART was better handled as a categorical variable. Time and its interactions with the other variables displayed nonlinear associations with total mean cost for the first 24 mo; thereafter trend was approximately linear. A restricted cubic spline (a cubic spline with linear tails) with three knots placed −4 to −1 mo, 4–7 mo, and 16–19 mo fitted the observed trends in our data; we experimented with the placement and number of knots using the Akaike Information Criteria and predictive plots to guide the final model selection. An interaction with the spline function for time was used for all variables except ART (first line versus second line) as the trend over time was difficult to quantify. Overall, the model was able to describe the trends in the data well, though in some intervals the trends in the baseline viral load and age variables were not well approximated at the extremes. While the main findings from this analysis using continuous variables did not differ from the previous analysis using categorical variables, some subtleties not previously shown were found in the relationships between costs and baseline CD4+ cell count and ART adherence. The association between baseline CD4+ cell count and mean total costs over time is shown in Figure 3; costs within each interval were compared with a referent group (CD4+ cell count = 200 cells/µl). Initially, the association with mean total cost followed a j-shape, with low CD4 counts associated with very high costs but also a modest increase in costs in patients with high counts. Over time the association between CD4+ cell count and mean total costs became less marked but costs were lowest for patients with higher CD4 counts. The association between ART adherence and mean total costs is shown in Figure 4; costs within each interval were compared with a referent group (ART adherence 75%). Initially, the model found three peaks: one around highly adherent patients, another around 50% adherence, and a smaller peak at very low adherence. Over time the lower peak fell away, the middle dominant peak moved to be centred at around 30% adherence, while the highly adherent patients were now associated with the lowest costs. Very low ART adherence was associated with low costs in all time intervals except in the peri-ART period. The association between mean total cost and baseline viral load (Figure S2) age at starting ART (Figure S3) are found in the supporting information (Figures S1–S3).

## Discussion

We analysed the direct health care costs of treating over 10,000 HIV-infected adults enrolled in a Southern African managed care ART programme with almost 600,000 patient months of follow-up, spanning 3 y before ART to 5 y on ART. We found a peak in costs in the period around the time of ART initiation, thereafter total mean costs dropped off to a plateau that persisted for 5 y. An important and novel feature of our study was the presentation of time-dependent associations between total mean costs and relevant variables. We identified lower baseline CD4+ cell count, higher baseline viral load, and shorter duration of CD4+ cell count monitoring before starting ART (as a proxy for HIV care) as being independently associated with higher costs in the early time periods. Lower ART adherence, being on second line ART, and starting ART at an younger age were most strongly associated with lower mean costs in later time periods, and the association with ART adherence became more marked over time.

The peak in costs in the peri-ART period we observed was largely driven by the high proportion of patients requiring hospitalisation. High rates of early morbidity, often resulting in hospitalisation or death, are characteristic of antiretroviral programmes in resource-limited settings. Patients on ART in low-income countries have higher early mortality compared with high-income countries, even after correcting for baseline differences in CD4+ cell counts [20]. A strength of our study is the analysis of cost data before starting ART. Few ART cost analyses include the period before starting ART. Higher costs in the first year on ART compared with later years with high rates of hospitalisation was reported in another South African study of a public sector ART programme, but they only assessed costs for 1 mo before starting ART and did not attempt to more accurately define the period of high cost [2]. Given our finding of high costs in the 4-mo period before starting ART, which was equivalent to 1.5 y of cost in patients on ART after the first year, other studies might have significantly underestimated the costs of providing HIV care just prior to starting ART.

We found that higher ART adherence was associated with lower costs particularly after removing antiretroviral drug costs. The magnitude of this association becomes greater as duration on ART increases. However, the continuous model showed that while highly adherent patients (>92%) were associated with the lowest total mean costs in later time intervals, they were associated with higher costs in the early time intervals. A similar association was found with high baseline CD4+ cell counts (>300 cells/µl) being associated with higher costs initially. These findings could be attributed to increased health-seeking behaviour leading to increased costs initially, but reduced costs over time. Very low ART adherence was associated with low total mean costs in all time intervals as these patients are presumably accessing minimal services. Our group has previously reported that ART adherence assessed by pharmacy refills in this cohort predicted both virological suppression [21] and survival [22]. Poor adherence limits the effectiveness of ART, drives resistance to first line regimens, and thus leads to earlier switching to costly second line ART. Despite the important role of ART adherence, existing economic models fail to include it.

Our analysis of the time-dependent associations with increased costs has several important public health implications. The high early costs of ART programmes could be reduced by starting ART at a CD4+ cell count of <350 cells/µl rather than <200 cells/µl (for patients without major symptomatic HIV disease). Our cohort does not allow for an evaluation of starting ART in patients with baseline CD4+ cell counts ≥350 cells/µl because AfA guidelines only allow these patients to start ART following an AIDS-defining illness or with other serious co-morbidity: costs were actually higher in this group compared with those starting ART with baseline CD4+ cell counts 200–349 cells/µl, presumably reflecting the costs of treating the morbidity that was the criterion for starting ART. The second intervention that could reduce early costs would be the earlier identification of HIV infection, illustrated by our finding that being in HIV care for more than 6 mo prior to starting ART reduced costs in the peri-ART period. The key driver of later costs with public health implications is ART adherence. Higher adherence prolongs time on the cheaper first line regimen, but also reduces non-ART direct costs in our study. The third intervention that might reduce costs would be to encourage ART programmes to invest in systems to monitor ART adherence and implement effective interventions if adherence is suboptimal. ART adherence could be monitored over short time periods of 3 to 4 mo, which identifies patients incurring higher costs and those at risk of virological failure [23].

We estimate that annual total direct health care costs are approximately US$2,400 (after the peak in costs in the peri-ART period) for patients accessing ART in the private sector. Lower costs were reported in two other South African studies. Harling reported costs of $2,502 in year one and $1,372 in year two of a donor-funded public sector program [24]. Rosen estimated the ART component of care to be US$757–US$1,126 in the first year of several different models of ART delivery to public sector patients, but non-ART–related clinic visits and hospitalisations were not included [25]. The incidence rate of hospitalisation we found in the first 6 mo on ART was similar to that reported in a South African public sector ART programme in the first 48 wk on ART, but our incidence was higher in later periods, which would increase costs [24]. Higher rates of hospitalisation in the private sector compared with the public sector after the initial period of ART probably reflect greater access in the private sector. Other factors driving higher costs in the private sector compared with public sector are higher costs for hospitalisation (US$340 versus US$202 per day, respectively) and viral load tests (US$62 versus US$42, respectively) [26].

Some findings of other ART programme cost studies differed from our analysis. We found that the ART component of costs was relatively small compared with other studies in resource-limited settings [2],[25],[27], which could be related to higher hospitalisation and other costs in our private sector setting. Younger age has been found to be associated with increased costs in some [28],[29], but not all studies [2]. We found a significant age effect with younger age (<25 y) associated with lower early but higher later costs and older age (≥50 y) associated with higher early and especially later costs. Finally, unlike the finding of another South African study [2], sex was not independently associated with costs, even after controlling for pregnancy-related costs and the higher proportion of men being on efavirenz. It is possible that our inclusion of ART adherence in our multiple regression model adjusted for sex differences, as we have previously shown that men have lower ART adherence than women [30].

There are a number of limitations to this analysis. First, our cohort consisted of private sector patients when the majority of patients in resource-limited settings are treated in the public sector. However, the baseline characteristics of our cohort (CD4+ cell count, proportion of females, and age) are comparable with cohorts from low-income countries [20],[31]. The BMI was <18.5 kg/m^2^ in 13% of our cohort compared with 19% in a South African public sector cohort [32], but their patients had more advanced disease as evidenced by their lower baseline CD4+ cell counts. These baseline nutritional differences would likely impact outcomes. We restricted our analysis to patients receiving NNRTI-based first line ART regimens, in keeping with WHO recommendations for resource-limited settings [8]. While we would not claim that our actual cost findings are generalisable to public sector settings or to other countries, we would argue that the variables that drive early and late costs are likely to be relevant even if the magnitude of the effect could differ.

Second, the impact of specific AIDS-defining illnesses on outcomes and costs was not included in this analysis because these data were not available. Third, as a provider's perspective was chosen for this analysis, the cost to society is not fully represented because we did not have data on direct non-health care costs and indirect costs. However, a provider's perspective is more appropriate for the aim of this study, which was to unpack the key drivers of health care costs in order to inform appropriate budgeting and planning. Fourth, the characteristics of the patients who left the scheme were different from those who remained, which may have affected our findings. However, there was no significant difference in the key baseline characteristic of CD4+ cell count and many of the other differences (e.g., age, difference of 0.1 log_10_ viral load) were small and are of questionable importance. Fifth, we chose to use the tariff amount as opposed to the amount claimed or reimbursed so that similar services would take the same monetary value and have further assumed that these tariffs are a suitable proxy for opportunity costs. While this could be a shortcoming, it is common to assume that market prices are a proxy for opportunity costs in economic evaluation given the difficulties in evaluating the latter [33]. Finally, cost minimisation should not be the only goal of health care providers, and other important aspects of care such as quality and outcomes are not addressed by our analysis.

In conclusion, we have described the temporal trends of costs of a large private sector HIV disease management programme in Southern Africa and shown that associations with costs change over time. Interventions that should reduce early costs include starting ART at higher CD4 counts and being in HIV care for longer periods before starting ART. Our results also indicate that systems to detect suboptimal ART adherence and interventions that improve adherence would reduce later costs. The increasing impact of ART adherence on costs over time suggests that this variable should be incorporated in economic models of ART.

## Supporting Information

Figure S1 Total monthly costs from 36 mo before starting ART to 60 mo on ART. Median and interquartile range, mean, and running-line least squares smooth are shown.
(0.15 MB TIF)

Figure S2 The proportional change in mean total monthly costs over time associated with baseline HIV viral load. Baseline HIV viral load was compared with the referent group (≥100,000 copies/ml) within each time interval from 4 mo before starting ART to 60 mo on ART with lighter blue indicating higher relative costs.
(0.31 MB TIF)

Figure S3 The proportional change in mean total monthly costs compared over time associated with age at starting ART. Age at ART was compared with the referent group (37 y) within each time interval from 4 mo before starting ART to 60 mo on ART with lighter blue indicating higher relative costs.
(0.40 MB TIF)

## Abbreviations

ART: antiretroviral therapy
NRTI: nucleoside reverse transcriptase inhibitor
NNRTI: non-nucleoside reverse transcriptase inhibitor
PI: protease inhibitor
