# Supplementary figures and images for: Early and Late Direct Costs in a Southern African Antiretroviral Treatment Programme: A Retrospective Cohort Analysis
Source: PLoS Med. 2009 Dec 1;6(12):e1000189. doi: 10.1371/journal.pmed.1000189 (PMC2777319; doi:10.1371/journal.pmed.1000189)

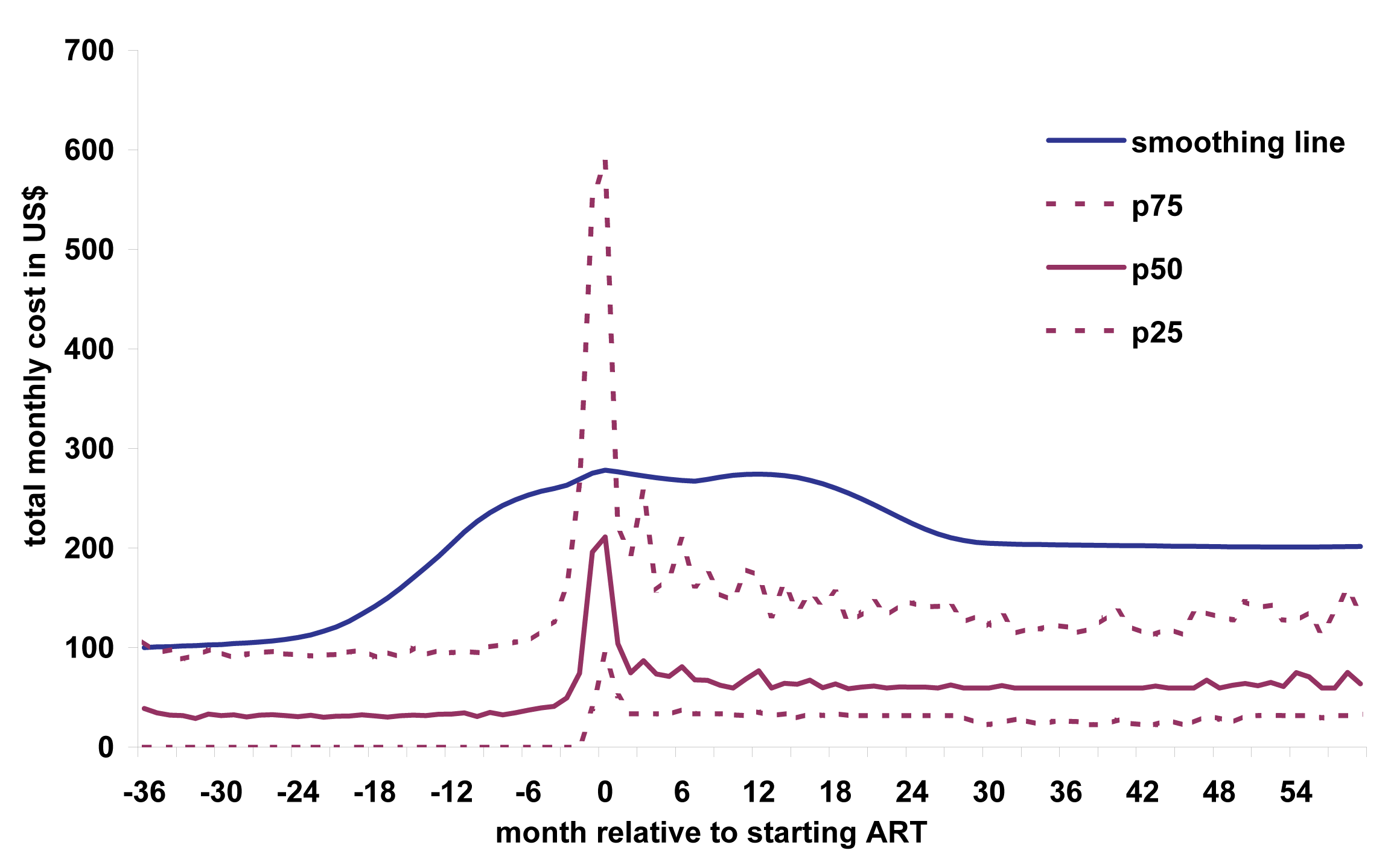

Supplement: Figure S1 — Total monthly costs from 36 mo before starting ART to 60 mo on ART. Median and interquartile range, mean, and running-line least squares smooth are shown. (0.15 MB TIF) [file pmed.1000189.s001.tif]

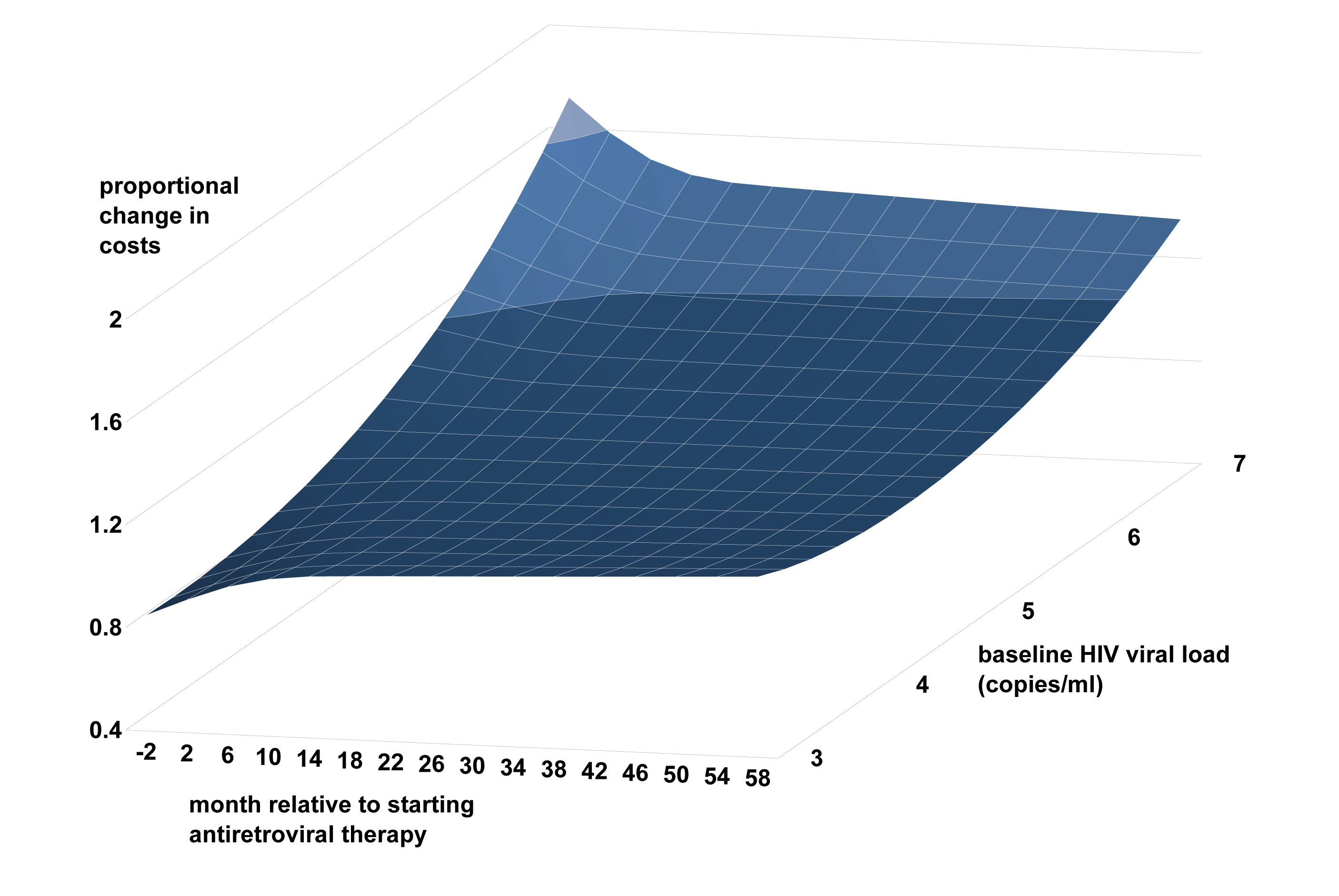

Supplement: Figure S2 — The proportional change in mean total monthly costs over time associated with baseline HIV viral load. Baseline HIV viral load was compared with the referent group (≥100,000 copies/ml) within each time interval from 4 mo before starting ART to 60 mo on ART with lighter blue indicating higher relative costs. (0.31 MB TIF) [file pmed.1000189.s002.tif]

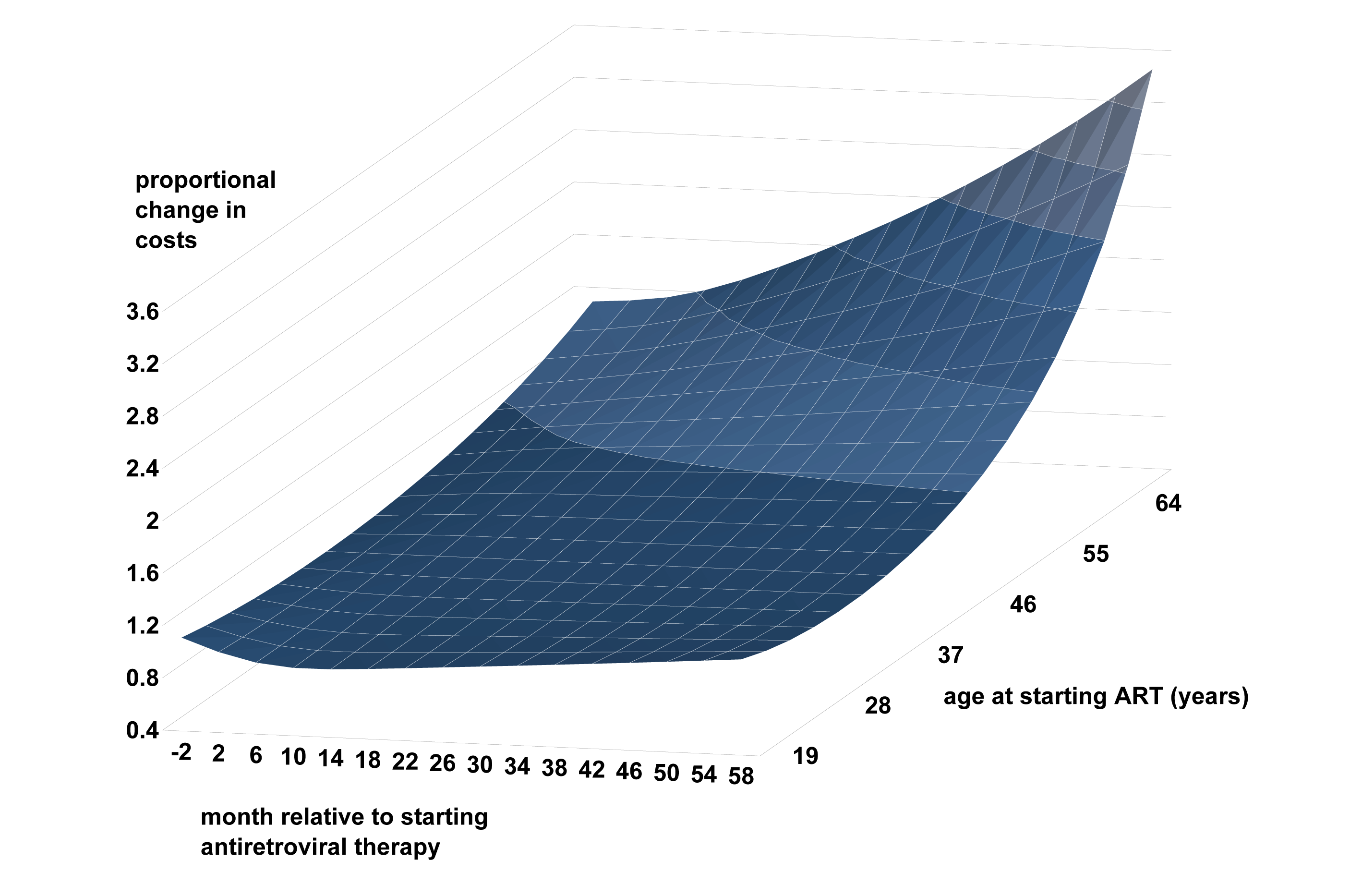

Supplement: Figure S3 — The proportional change in mean total monthly costs compared over time associated with age at starting ART. Age at ART was compared with the referent group (37 y) within each time interval from 4 mo before starting ART to 60 mo on ART with lighter blue indicating higher relative costs. (0.40 MB TIF) [file pmed.1000189.s003.tif]
